# World species of the genus *Platyscelio* Kieffer (Hymenoptera: Platygastridae)

**DOI:** 10.3897/zookeys.50.485

**Published:** 2010-06-30

**Authors:** Charuwat Taekul, Norman F. Johnson, Lubomír Masner, Andrew Polaszek, Rajmohana K.

**Affiliations:** 1 Department of Evolution, Ecology and Organismal Biology, The Ohio State University, 1315 Kinnear Road, Columbus, Ohio 43212, U.S.A.; 2 Agriculture and Agri-Food Canada, K.W. Neatby Bldg., Ottawa, Ontario K1A 0C6, Canada; 3 Department of Entomology, The Natural History Museum, Cromwell Road, London SW7 5BD, UK; 4 Zoological Survey of India, Western Ghats Field Research Station, Jafarkhan Colony, Calicut – 673006, Kerala, India

**Keywords:** Platygastridae, Scelioninae, biodiversity informatics, egg parasitoids, Tettigoniidae

## Abstract

The genus Platyscelio Kieffer (Hymenoptera: Platygastridae, Scelioninae) is a widespread group in the Old World, found from West Africa to northern Queensland, Australia. The species concepts are revised and a key to world species is presented. The genus is comprised of 6 species, including 2 known species which are redescribed: Platyscelio africanus Risbec (Benin, Cameroon, Central African Republic, Ghana, Guinea, Guinea-Bissau, Ivory Coast, Kenya, Mozambique, Nigeria, Sierra Leone, South Africa, Tanzania, Togo, Uganda, Yemen, Zimbabwe); and Platyscelio pulchricornis Kieffer (Australia, Bangladesh, China, India, Indonesia, Japan, Malaysia, Papua New Guinea, Philippines, Solomon Islands, Taiwan, Thailand, Vanuatu, Vietnam). Five species-group names are considered to be junior synonyms of Platyscelio pulchricornis: Platyscelio abnormis Crawford **syn. n.**, Platyscelio dunensis Mukerjee **syn. n.**, Platyscelio mirabilis Dodd **syn. n.**, Platyscelio punctatus Kieffer **syn. n.**, and Platyscelio wilcoxi Fullaway. The following species are hypothesized and described as new taxa: Platyscelio arcuatus Taekul & Johnson, **sp. n.** (Western Australia); Platyscelio mysterium Taekul & Johnson, **sp. n.** (Zimbabwe, Botswana, South Africa); Platyscelio mzantsi Taekul & Johnson, **sp. n.** (South Africa); and Platyscelio striga Taekul & Johnson, **sp. n.** (Western Australia).

## Introduction

Species of Platyscelio (Hymenoptera: Platygastroidea, Platygastridae) are morphologically unique among the known Scelioninae by a number of characters, most distinctively the extremely flat body, the broad hypostomal bridge, and the absence of a netrion. The genus was originally described by Kieffer (1905) with a single species, Platyscelio pulchricornis from Dilo in British New Guinea. To date, six species-group taxa have been described. Five species are recorded in Asia, Australia and Oceania: Platyscelio pulchricornis Kieffer, Platyscelio abnormis Crawford, Platyscelio dunensis Mukerjee, Platyscelio mirabilis Dodd, and Platyscelio punctatus Kieffer. Only one species is known from Africa, Platyscelio africanus Risbec, described from Cameroon.

Platyscelio wasclassified within the subfamily Scelioninae of the family Scelionidae by Kieffer (1905), but was not placed in any tribe until Kozlov (1970) erected the monobasic tribe Platyscelionini. Sharkey (2007) has subsequently combined the families Scelionidae and Platygastridae under the single name Platygastridae, but he did not address the status of their respective subfamilies or tribes. Kozlov (1970) asserted that Platyscelionini is close to the tribe Scelionini, but did not substantiate this hypothesis. Masner (1976) noted that the genus is distant from other Scelioninae due to its lack of a netrion, the greatly reduced palpi, and the expansion of the female antennal scape into a flat, almost triangular piece armed laterally with a sharp spine. Austin and Field (1997) examined the ovipositor structure and concluded that Platyscelio possesses a Scelio-type system. They also commented on two unusual features of the genus: the second gonocoxae are developed as broad membranous plates, and the lateral apodemes of the sixth metasomal sternum in the female protrude proximally past the telescopic tube at rest. Platyscelio was not included as a taxon in the most comprehensive attempt to infer relationships within the Platygastroidea published to date (Murphy et al. 2007).

The known hosts of species in the subfamilies Scelioninae, Teleasinae, and Telenominae are the eggs of insects and spiders (Austin et al. 2005). In the original description of Platyscelio africanus, Risbec (1956) mentioned that the 15 females and 3 males were obtained from eggs of “Locustidae.” Specimens of Platyscelio pulchricornis from the National Museum of Natural History (Washington, DC; OSUC 207839) include unidentified host eggs (Figs 73, 74). Two reared series of Platyscelio pulchricornis in the Natural History Museum, London are from eggs identified as “?Conocephalus sp” collected from sugarcane (BMNH(E)#790205) and rice in Papua New Guinea (BMNH(E)#790194) (Figs 75, 76). Agyen-Sampong (1980, cited in Heinrichs and Barrion 2004) reported Platyscelio sp. as a parasitoid of Conocephalus conocephalus (Linnaeus) (Orthoptera: Tettigoniidae). Agyen-Sampong conducted all his observations in West Africa, mostly Sierra Leone. These data, albeit fragmentary, suggest that Conocephalus eggs are at least among the hosts of Platyscelio spp, across the range of the genus from West Africa to Australasia.

Kozlov (1970) asserted that the host of the genus must be the flattened eggs of Phaneropterinae (Orthoptera: Tettigoniidae), and also suggested that the strongly flattened body of Platyscelio may indicate that the species are phoretic in habit.

In more than a century since its original description, Platyscelio has never been comprehensively reviewed or revised. Our goal of this paper is to present a taxonomic revision of the world species of the genus Platyscelio, as well as to expand the biogeographic data associated with these species. The taxonomic history of the genus is summarized and existing concepts are reviewed. Four new species are proposed, two from Western Australia, two from southern Africa.

## Materials and methods

This work is based upon specimens in the following collections, with abbreviations used in the text: AEIC, American Entomological Institute, Gainesville, FL ^1^; ANIC, Australian National Insect Collection, Canberra, Australia ^2^; BMNH, The Natural History Museum, London, UK ^3^; CASC, California Academy of Sciences, San Francisco, CA ^4^; CNCI, Canadian National Collection of Insects, Ottawa, Canada ^5^; EMEC, Essig Museum of Entomology, Berkeley, CA ^6^; ISNB, Institut Royal des Sciences Naturelles de Belgique, Bruxelles, Belgium ^7^; MCSN, Museo Civico di Storia Naturale “Giacomo Doria”, Genova, Italy ^8^; MZLU, Lund University, Lund, Sweden ^9^; NZSI, Zoological Survey of India, North Regional Station, Uttaranchal, India ^10^; OSUC, C.A. Triplehorn Insect Collection, Columbus, OH ^11^; RMNH, Nationaal Natuurhistorisch Museum, Leiden, Netherlands ^12^; ROME, Royal Ontario Museum, Toronto, Canada ^13^; SAMA, South Australian Museum, Adelaide, Australia ^14^; SAMC, Iziko Museums of South Africa, Cape Town, South Africa ^15^; SANC, South African National Collection of Insects, Pretoria, South Africa ^16^; SCAU, South China Agricultural University, Guangzhou, China ^17^; TARI, Taiwan Agricultural Research Institute - Entomology, Taichung, Taiwan ^18^; UASK, Ukrainian Academy of Sciences, Kiev, Ukraine ^19^; UCDC, University of California, Davis, CA ^20^; USNM, National Museum of Natural History, Washington, DC ^21^; WINC, Waite Insect and Nematode Collection, Adelaide, Australia ^22^; ZMAS, Zoological Museum, Academy of Sciences, St. Petersburg, Russia ^23^.

Abbreviations and morphological terms used in text: A1, A2, ... A12antennomere 1, 2, … 12; claval formuladistribution of the large, multiporous basiconic sensilla on the underside of apical antennomeres of the female, with the segment interval specified followed by the number of sensilla per segment (Bin 1981); POLposterior ocellar line, the shortest distance between inner margins of posterior ocelli; OOLocular ocellar line, the shortest distance from inner orbit and outer margin of lateral ocellus (Masner 1980); T1, T2, ... T7metasomal tergite 1, 2, ... 7. Morphological terminology otherwise follows Masner (1980) and Mikó et al. (2007).

In the Material Examined the numbers prefixed with “OSUC” are unique identifiers for the individual specimens. The label data for all specimens have been georeferenced and recorded in the Hymenoptera On-Line database, and details on the data associated with these specimens can be accessed at the following link, purl.oclc.org/NET/hymenoptera/hol, and entering the identifier in the form. Note the space between the acronym and the number.

Data associated with the genus Platyscelio can be accessed at http://hol.osu.edu/index.html?id=543. Species descriptions were generated using a database application, vSysLab ^24^, designed to facilitate the production of a taxon by character data matrix, and to integrate those data with the existing taxonomic and specimen-level database. Data may be exported in both text format and as input files for other applications. The text output for descriptions is in the format of “Character: Character state (s). Images and measurements were made using AutoMontage and Cartograph extended-focus software, using JVC KY-F75U digital camera, Leica Z16 APOA microscope, and 1X objectve lens. Images are achived at Morphbank and in Specimage^25^, the image database at The Ohio State University.

In this article we have followed the precedent of Pyle et al. (2008) and Johnson et al. (2008) in the implementation of biodiversity informatics standards within a taxonomic publication. The electronic version of the paper contains hyperlinks to external resources. Insofar as possible the external information conforms to standards developed and maintained through the organization Biodiversity Information Standards^26^ (Taxonomic Database Working Group). All new species have been prospectively registered with Zoobank (Polaszek et al. 2005), and other taxonomic names, where appropriate, have been retrospectively registered. The external hyperlinks are explicitly cited in the endnotes so that users of the printed version of this article have access to the same resources. Life sciences identifiers, LSIDs, may be resolved at the specified URLs or at lsid.tdwg.org.

This work is conducted as part of the Platygastroidea Planetary Biodiversity Inventory. The contributions of the authors are as follows: C. Taekul: character definition, species concept development, imaging, measurement, key development, manuscript preparation; N.F. Johnson: character definition, generic concept development, species concept development, imaging, key development, manuscript preparation; L. Masner: generic concept development, species concept development, character definition; A. Polaszek: species concept and key testing, imaging, manuscript preparation; Rajmohana K.: key testing, comparison of holotype of Platyscelio dunensis Mukerjee, imaging.

## Platyscelio

Kieffer

urn:lsid:zoobank.org:act:003FB0A7-9A8B-4670-B481-5108C3044595

urn:lsid:biosci.ohio-state.edu:osuc_concepts:543

Platyscelio Kieffer 1905:11 Original description. Type: Platyscelio pulchricornis Kieffer, by monotypy. Brues 1908: 27, 40 (diagnosis, list of species, keyed); Kieffer 1908: 113 (keyed); Kieffer 1910: 62, 66 (description, list of species, keyed); Dodd 1913: 130 (keyed); Kieffer 1913: 222 (description); Brues 1922: 21 (diagnosis, figure); Kieffer 1926: 265, 553 (description, keyed, key to species); Mani 1941: 29 (catalog of species of India); Muesebeck and Walkley 1956: 386 (citation of type species); Baltazar 1966: 186 (catalog of species of the Philippines); Masner 1976: 10, 55 (description, keyed); Mani and Sharma 1982: 190 (description); Galloway and Austin 1984: 9, 75 (diagnosis, keyed); Johnson 1992: 461 (catalog of world species); Austin and Field 1997: 34, 68 (structure of ovipositor system, discussion of phylogenetic relationships); Lê 2000: 31 (keyed); Rajmohana K. 2006: 117, 128 (description, keyed); Kononova and Kozlov 2008: 25, 319 (description, keyed).

### Diagnosis.

Platyscelio is distinguished from most other genera of Scelioninae sensu Masner (1976) by the strongly dorsoventrally depressed, flat, and foliaceous body. A small number of other groups, such as Aradophagus Ashmead and Synoditella Muesebeck, are also more or less strongly depressed. Platyscelio may be distinguished from them by the absence of a netrion; the mandibles are tridentate, with the middle tooth small; the postmarginal vein is very short or absent, clearly shorter than the stigmal vein; and vein R in the hind wing is complete, extending to the hamuli on the costal margin of the wing.

### Description.

Moderate-sized, length 3.0–5.6 mm; head prognathous, flattened anteroposteriorly, mesosoma and metasoma strongly dorsoventrally depressed; body black; macropterous.

#### Head:

Head in dorsal view strongly transverse; vertex laterad of posterior ocellus smooth or with few faint striae, between posterior ocelli finely longitudinally striate; hyperoccipital carina present as fine ridge on vertex between compound eyes; occipital carina absent; posterior ocellus distinctly separated from inner orbit of compound eyes, OOL > diameter of ocellus; compound eye large, appearing glabrous; frons without depression, shallowly convex, with median longitudinal sulcus bifurcating dorsally near median ocellus and ventrally near toruli; interantennal process well-developed, narrow; torular triangle present; submedian carina sometimes present; orbital carina sometimes present; lower frons, including cheek, with weak fanlike striae arising from mandibular condyle; shortest distance on frons between eyes less than eye height; inner orbits weakly diverging ventrally; postclypeus strongly projecting above anteclypeus, subtriangular, anteclypeus short, longest medially, lateral corners not produced; malar sulcus present; gena variably expanded, smooth to longitudinally striate or with few faint striae; labrum hidden by clypeus; mandible short, apex tridentate, middle tooth distinctly shortest, teeth arranged transversely; maxillary palpus 2-segmented, all segments cylindrical; labial palpus 1-segmented, very short; antenna 12-merous in both sexes; radicle very broad, inserted into ventral apex of A1, nearly parallel to longitudinal axis of A1, with small lateral spine; A1 almost triangular and expanded outwardly into spine, particularly in female; A2 distinctly shorter than A3; gustatory sensilla on female antenna arranged in longitudinal pairs on apical antennomeres; clava laterally compressed, claval formula A12–A8: 1-2-2-2-1; male antenna with tyloid on A5.

#### Mesosoma:

Mesosoma in dorsal view longer than wide, in lateral view much longer than high; pronotum in dorsal view strongly narrowed laterally, anterolateral corner weakly angulate; transverse pronotal carina absent; vertical epomial carina absent; dorsal epomial carina absent; lateral face of pronotum concave; netrion absent; anterior margin of mesoscutum strongly flexed ventrally to meet pronotum; mesoscutum semicircular in outline, posterolateral corner rounded; parapsidal line variably developed; notaulus variably developed: absent, nearly percurrent or percurrent, mesoscutum sometimes with strong sublateral carina paralleling notaulus; skaphion absent; prespecular sulcus and posterior mesepimeral sulcus present; speculum smooth, rarely longitudinally striate (Platyscelio arcuatus) transscutal articulation well developed; mesoscutellum rectangular in outline, truncate posteriorly, sculpture smooth to longitudinally striate; axilla small; posterior scutellar sulcus interrupted medially or complete; metanotum narrow, metascutellum clearly differentiated, size of metascutellum variable; dorsal surface of propodeum variable, weakly setose posteriorly; median propodeal sulcus present; plicae well developed; propodeal projections reduced; sternaulus absent; metapleural pit reduced; metapleural sulcus present dividing metapleuron into dorsal and ventral areas; setation of posterior half of ventral metapleural area variable; legs elongate; posterior surface of hind coxa smooth, glabrous to densely setose; trochantellus present; tibial spur formula 1-1-1; tarsal formula 5-5-5; tarsomeres cylindrical, broadening apically; pretarsal claw simple; apex of fore wing extending to or slightly beyond apex of S5 in female and S6 in male, hyaline or infuscate; R straight, extending slightly beyond basal half of length of wing, without strong, elongate bristles, gradually approaching costal margin apically, contiguous with costal margin for distance clearly exceeding length of r-rs (i.e., marginal vein elongate); r-rs straight; R1 usually absent, reduced, stumplike in some species (Platyscelio arcuatus and Platyscelio mzantsi) (i.e., postmarginal vein absent); bulla absent; Rs+M (basal vein) weakly to clearly indicated, nebulous; hind wing with R extending from base of wing to hamuli; three hamuli present.

#### Metasoma:

Metasoma elongate, parallel-sided, strongly flattened dorsoventrally; female with six visible terga and sterna, male with seven visible terga and sterna; second to fifth segments equal in length, third and fourth widest, subequal to each other in size; submarginal ridge developed, defined by narrow laterotergites to form deep submarginal rim; sublateral carina well developed on tergites, rarely absent (Platyscelio striga); no spiracles visible; sculpture on T1–T4 variable; S1 not laterally compressed; felt field absent.

### Link to distribution map. ^27^

The genus Platyscelio is a widespread group throughout the Old World tropics, extending from West Africa to Queensland. Four species have relatively restricted distributions: Platyscelio arcuatus and Platyscelio striga (Western Australia), Platyscelio mysterium (Zimbabwe, Botswana, South Africa), and Platyscelio mzantsi (South Africa). Two species are widespread: Platyscelio africanus occurs in the Afrotropical realm from Senegal east to Yemen and south to South Africa; and Platyscelio pulchricornis is found in India and Southeast Asia east to Papua New Guinea and eastern Australia.

### Key to species of Platyscelio

**Table:** 

1	Striae within ocellar triangle sparse (fewer than 20); distance between anterior ocellus and posterior ocellar line in frontal view greater than or equal to 0.5× POL (Figs. 17, 71)	2
-	Striae within ocellar triangle dense (more than 20); distance between anterior ocellus and posterior ocellar line in frontal view less than 0.5× POL (Figs. 5, 11, 35, 41, 47, 59, 65)	3
2	Sculpture on T1 longitudinally striate, interstices with coriaceous microsculpture (Fig. 18); postmarginal vein absent (Fig. 16); notaulus with mesal margin arched, lateral margin straight (Fig. 15); legs beyond coxae yellow (Figs. 13, 14); female outer lateral apex of scape sharply pointed (Fig. 17) (Western Australia)	Platyscelio arcuatus Taekul & Johnson, sp. n.
-	Sculpture on T1 longitudinally striate throughout (Fig. 72); postmarginal vein present, stumplike (Fig. 70); notaulus with both mesal and lateral margins arched (Fig. 69); legs beyond coxae brown (Figs. 67, 68); female outer lateral apex of scape bluntly rounded (Fig. 71) (South Africa)	Platyscelio mzantsi Taekul & Johnson, sp. n.
3	Frontal sculpture between inner orbit and central keel longitudinally striate, striae either extending through most of length of frons, or with few (4–5) striae restricted to upper half of frons (Figs. 23, 65)	4
-	Frons between inner orbit and central keel smooth (Figs. 5, 11, 29, 35, 41, 47)	5
4	Mesoscutum with one lateral carina; notaulus absent (Fig. 21); sculpture on T1 longitudinally striate laterally, uniformly setigerous punctate medially (Fig. 22) (Botswana, South Africa, Zimbabwe)	Platyscelio mysterium Taekul & Johnson, sp. n.
-	Mesoscutum with two lateral carinae; notaulus present (Fig. 63); sculpture on T1 longitudinally striate throughout (Fig. 64) (Western Australia)	Platyscelio striga Taekul & Johnson, sp. n.
5	Posterior scutellar sulcus complete (Figs. 3, 6, 9, 12); margin of propodeum longitudinally striate laterally, rugulose posteriorly (Figs. 6, 12); vertex between inner orbit and posterior ocellus densely striate (Figs. 5, 11) (sub-Saharan Africa, Yemen)	Platyscelio africanus Risbec
-	Posterior scutellar sulcus interrupted medially (Figs. 27, 33, 39, 45, 57); margin of propodeum smooth laterally, longitudinally striate to rugulose posteriorly (Figs. 27, 33, 40, 48, 53, 57); vertex between inner orbit and posterior ocellus smooth (Figs. 29, 35, 41, 47, 51, 59) (India east to Guam, Solomon Islands, Vanuatu, eastern Australia)	Platyscelio pulchricornis Kieffer

### Platyscelio africanus

Risbec

urn:lsid:zoobank.org:act:0814CE3A-F4CF-434C-98AA-8E882FA2F202

urn:lsid:biosci.ohio-state.edu:osuc_concepts:5089

[Fig Figs01-06] Figures 1-12 Morphbank^28^

Platyscelio africanus Risbec 1956: 105 (original description); Masner 1976: 55 (description).

#### Description.

##### General:

Body length of male: 2.95–4.35 mm (n=20). Body length of female: 3.46–4.28 mm (n=20).

##### Head:

Length between anterior ocellus and posterior ocellar line in frontal view: less than 0.5 times POL. Striae within ocellar triangle: dense (greater than 20). Vertex sculpture between inner orbit and posterior ocellus: densely striate. Frontal sculpture between inner orbit and central keel: smooth. Submedial ventral area of head anterior to fossa: smooth, finely longitudinally striate posteriorly. Orbital carina: absent. Sculpture of malar region: smooth, faintly longitudinal striae with limited microsculpture near eye.

##### Antenna:

Color of female antenna: A1–A7 yellow to light brown, A8–A12 dark brown to black. Female outer lateral apex of scape: sharply pointed. Claval shape: apical margin of A9–A11 concave, closely fitting basal margin of following antennomere. Color of male antenna: yellow to light brown throughout.

##### Mesosoma:

Sculpture on medial lobe of mesoscutum: longitudinally striate with elongate punctures. Setation of medial lobe of mesoscutum: moderately dense, even. Notaulus: present. Notaulus form: mesal and lateral margin arched. Length of notaulus: abbreviated, clearly not reaching anterior margin of mesoscutum. Width of notaulus anteriorly: parallel-sided. Pilosity of notaulus: absent. Number of lateral carinae on mesoscutum: 0. Medial carina of mesoscutum: absent. Parapsidal line: present. Posterior scutellar sulcus: complete. Setation of posterior half of ventral metapleural area: sparse (less than 25 setae). Metascutellum size: wide, metanotum lateral to metascutellum reduced, with 0–3 foveae. Sculpture on ventral metapleural area: longitudinally striate or with few reticulations. Median propodeal sulcus: widened posteriorly. Sculpture of submedian propodeal field: longitudinally striate. Posterolateral margin of propodeum: longitudinally striate laterally, rugulose posteriorly. Color of legs: coxae dark brown to black, otherwise yellow throughout, rarely hind femur dark brown.

##### Wings:

Female Postmarginal vein: absent. Fore wing: hyaline.

##### Metasoma:

Sculpture on T1: longitudinally striate with setigerous punctures medially. Sublateral carina on T2–T4: present anteriorly, absent posteriorly. Sculpture on T2–T4: setigerous punctures throughout, longitudinally striate anteriorly.

**Figures 1–6. Figs01-06:**
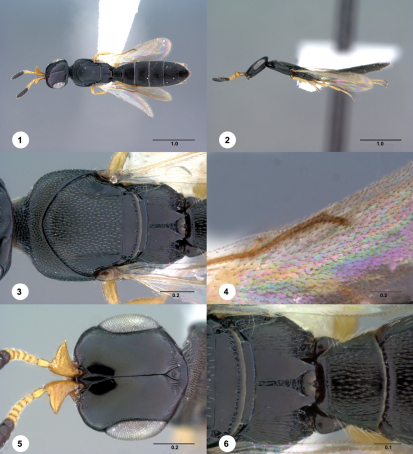
^80^ Platyscelio africanus Risbec, female (OSUC 250659). 1 Dorsal habitus 2 Lateral habitus 3 Mesosoma, dorsal view 4 Fore wing marginal vein, dorsal view 5 Head, dorsal view 6 Mesoscutellum, propodeum and T1, dorsal view. Scale bars in millimeters.

**Figures 7–12.**
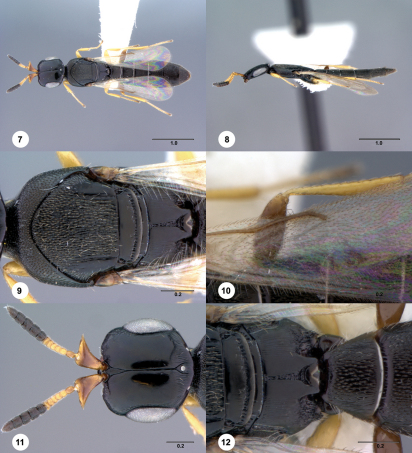
^81^ Platyscelio africanus Risbec, female (OSUC 207985). 7 Dorsal habitus 8 Lateral habitus 9 Mesosoma, dorsal view 10 Fore wing marginal vein, dorsal view 11 Head, dorsal view 12 Mesoscutellum, propodeum and T1, dorsal view. Scale bars in millimeters.

#### Diagnosis.

Platyscelio africanus is similar to Platyscelio pulchricornis in the lack of sculpture on the frons between the inner orbit and the central keel. Itmay be distinguished by the complete posterior scutellar sulcus, and the sculpture on the margin of the propodeum is longitudinally striate laterally and rugulose posteriorly.

#### Link to distribution map. ^29^

#### Material examined.

Holotype female: CAMEROON: Garoua (deposited in MNHN). Other material: (78 females, 57 males) BENIN: 14 females, 5 males, CASENT 2137991 (CASC); OSUC 207951-207968 (CNCI). CAMEROON: 17 females, 7 males, BMNH(E)#790211, 848520-848536 (BMNH); CASENT 2137986 (CASC); OSUC 250657-250661 (CNCI). CENTRAL AFRICAN REPUBLIC: 1 female, 1 male, OSUC 176086, 247778 (SAMC). GHANA: 2 males, BMNH(E)#790201, 848510 (BMNH). GUINEA: 1 female, 1 male, OSUC 207895, 250625 (CNCI). GUINEA-BISSAU: 1 female, OSUC 253728 (MZLU). IVORY COAST: 22 females, 13 males, OSUC 207977-207983, 207986-208007 (CNCI); OSUC 58731-58736 (OSUC). KENYA: 2 females, 7 males, OSUC 173854, 207969-207976 (CNCI). MOZAMBIQUE: 3 males, OSUC 207948-207950 (CNCI). NIGERIA: 9 females, 5 males, BMNH(E)#790200 (BMNH); OSUC 250639-250650 (CNCI); OSUC 173856 (OSUC). SIERRA LEONE: 2 females, 5 males, BMNH(E)#790195, 848506 (BMNH); OSUC 253722-253726 (MZLU). SOUTH AFRICA: 2 females, 5 males, OSUC 207938, 207943-207946, 250663 (CNCI); OSUC 253727 (MZLU). TANZANIA: 3 females, 1 male, OSUC 253741-253744 (SAMC). TOGO: 1 female, OSUC 253754 (CNCI). UGANDA: 1 female, 1 male, OSUC 207984-207985 (CNCI). YEMEN: 1 female, 1 male, OSUC 250651-250652 (CNCI). ZIMBABWE: 1 female, BMNH(E)#790209 (BMNH).

#### Comments.

This species is widespread in the Afrotropical realm, extending from east Africa to Yemen and south to the north of South Africa. The color of the female antenna is variable: the scape is yellow to light brown, but in some specimens is dark brown to black (OSUC 207985, 207972; Figs 7, 11). The color variability is also seen on the legs: coxae are dark brown to black, otherwise the legs are yellow throughout in most specimens, but in some the hind femur is dark brown (OSUC 207985, 207954, 207955).

### Platyscelio arcuatus

Taekul & Johnson sp. n.

urn:lsid:zoobank.org:act:E6605CB9-43A7-4B8D-A4C4-89B9049B9677

urn:lsid:biosci.ohio-state.edu:osuc_concepts:242615

**Figures 13–18.**
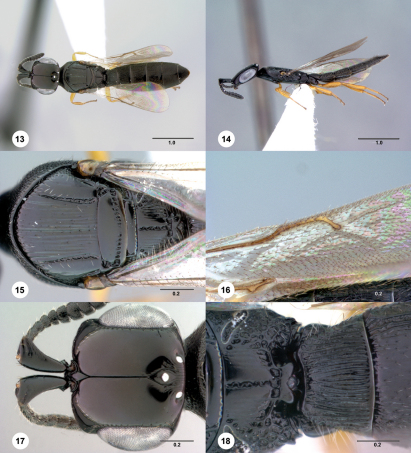
^82^ Platyscelio arcuatus, sp. n., holotype female (OSUC 250635). 13 Dorsal habitus 14 Lateral habitus 15 Mesosoma, dorsal view 16 Mesosoma, lateral view 17 Head, dorsal view 18 Propodeum and T1, dorsal view. Scale bars in millimeters.

Figures 13–18 Morphbank^30^ 

#### Description.

##### General:

Body length of male: 3.87 mm (n=1). Body length of female: 3.48–3.97 mm (n=3).

##### Head:

Length between anterior ocellus and posterior ocellar line in frontal view: greater than or equal to 0.5 times POL. Striae within ocellar triangle: sparse (equal to or less than 20). Vertex sculpture between inner orbit and posterior ocellus: smooth or with few faint striae. Frontal sculpture between inner orbit and central keel: smooth. Submedial ventral area of head anterior to fossa: longitudinally striate throughout. Orbital carina: absent. Sculpture of malar region: longitudinally striate or with few faint striae.

##### Antenna:

Color of female antenna: dark brown to black throughout. Female outer lateral apex of scape: sharply pointed. Claval shape: apical margin of A9–A11 concave, closely fitting basal margin of following antennomere. Color of male antenna: dark brown to black throughout.

##### Mesosoma:

Sculpture on medial lobe of mesoscutum: longitudinally striate. Setation of medial lobe of mesoscutum: sparse to glabrous. Notaulus: present. Notaulus form: mesal margin arched, lateral margin straight. Length of notaulus: percurrent or nearly so. Width of notaulus anteriorly: narrowed anteriorly. Pilosity of notaulus: present. Number of lateral carinae on mesoscutum: 0. Medial carina of mesoscutum: present. Parapsidal line: present. Posterior scutellar sulcus: interrupted medially or complete. Setation of posterior half of ventral metapleural area: sparse (less than 25 setae). Metascutellum size: narrow, metanotum lateral to metascutellum with 4–6 foveae. Sculpture on ventral metapleural area: longitudinally striate, coriaceous microsculpture within interstices or strongly reticulate rugose with foveolae. Median propodeal sulcus: narrow throughout length. Sculpture of submedian propodeal field: longitudinally striate, in male interstices with coriaceous microsculpture. Posterolateral margin of propodeum: reticulate rugose with foveolae throughout. Color of legs: coxae dark brown to black, otherwise yellow throughout.

##### Wings:

Postmarginal vein: reduced, stumplike. Fore wing: hyaline.

##### Metasoma:

Sculpture on T1: longitudinally striate, coriaceous microsculpture within interstices. Sublateral carina on T2–T4: present, percurrent. Sculpture on T2–T4: longitudinally striate with coriaceous microsculpture within interstices.

#### Diagnosis.

Platyscelio arcuatus may be separated from Platyscelio striga (also from Western Australia) by the less densely striate sculpture within the ocellar triangle (20 striae or fewer).

#### Etymology.

The epithet arcuatus, Latin for bent like a bow, refers to the shape of the notaulus.

#### Link to distribution map. ^31^

#### Material examined.

Holotype female: AUSTRALIA: Western Australia, Keystone Rd., 3 km W Walpole, 34°59.01'S 116°40.76'E, George, Hawks, Munro, YPT, OSUC 250635 (deposited in ANIC). Paratypes: AUSTRALIA: 3 females, 1 male, OSUC 250633–250636 (CNCI).

### Platyscelio mysterium

Taekul & Johnson sp. n.

urn:lsid:zoobank.org:act:9323A442-6603-4C4E-A1E4-5A48F7A291A4

urn:lsid:biosci.ohio-state.edu:osuc_concepts:242616

**Figures 19–24.**
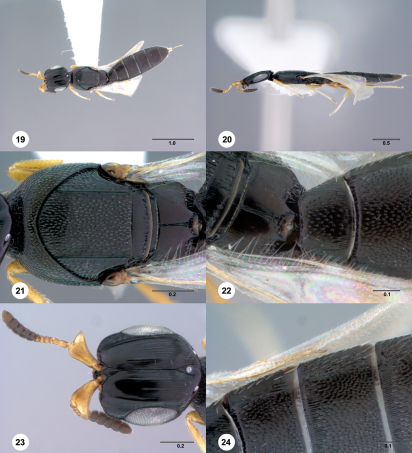
^83^ Platyscelio mysterium, sp. n., holotype female (OSUC 171372). 19 Dorsal habitus 20 Lateral habitus 21 Mesosoma, dorsal view 22 Mesosoma, lateral view 23 Head, dorsal view 24 Sublateral carina on T2–T4, dorsal view. Scale bars in millimeters.

Figures 19–24 Morphbank^32^ 

#### Description.

##### General:

Body length of male: 3.46–4.20 mm (n=7). Body length of female: 3.14–4.24 mm (n=8).

##### Head:

Length between anterior ocellus and posterior ocellar line in frontal view: less than 0.5 times POL. Striae within ocellar triangle: dense (greater than 20). Vertex sculpture between inner orbit and posterior ocellus: densely striate. Frontal sculpture between inner orbit and central keel: longitudinally striate, striae extending through most of length of frons. Submedial ventral area of head anterior to fossa: smooth, finely longitudinally striate posteriorly. Orbital carina: present. Sculpture of malar region: longitudinally striate or with few faint striae.

##### Antenna:

Color of female antenna: A1–A7 yellow to light brown, A8–A12 dark brown to black. Female outer lateral apex of scape: sharply pointed. Claval shape: apical margin of A9–A11 concave, closely fitting basal margin of following antennomere. Color of male antenna: brown or dark brown to black throughout.

##### Mesosoma:

Sculpture on medial lobe of mesoscutum: longitudinally striate with elongate punctures. Setation of medial lobe of mesoscutum: moderately dense, even. Notaulus: absent. Pilosity of notaulus: absent. Number of lateral carinae on mesoscutum: 1. Medial carina of mesoscutum: absent. Parapsidal line: present. Posterior scutellar sulcus: complete. Setation of posterior half of ventral metapleural area: sparse (less than 25 setae). Metascutellum size: wide, metanotum lateral to metascutellum reduced, with 0–3 foveae. Sculpture on ventral metapleural area: smooth anteriorly, coarsely foveolate punctate posteriorly. Median propodeal sulcus: narrow throughout length. Sculpture of submedian propodeal field: smooth throughout or with few faint striae. Posterolateral margin of propodeum: smooth laterally, longitudinally striate to rugulose posteriorly. Color of legs: coxae dark brown to black, otherwise variable.

##### Wings:

Postmarginal vein: absent. Fore wing: hyaline.

##### Metasoma:

Sculpture on T1: longitudinally striate laterally, uniformly setigerous punctate medially. Sublateral carina on T2–T4: present anteriorly, absent posteriorly. Sculpture on T2–T4: setigerous punctures throughout, longitudinally striate anteriorly.

#### Diagnosis.

Platyscelio mysterium is distinguished from other species by the presence of only a single lateral carina on the mesoscutum, the lack of a notaulus, and the presence of orbital carinae on the frons (Figs 21, 23).

#### Etymology.

The epithet mysterium, Latin for mystery, refers to the interpretation of the mesoscutal carinae.

#### Link to distribution map. ^33^

#### Material examined.

Holotype female: BOTSWANA: Serowe, Farmer’s Brigade, 22°22.998'S, 026°43.002'E, May 1989, Malaise trap, P. Forchhammer, OSUC 171372 (deposited in USNM). Paratypes: (11 females, 14 males) BOTSWANA: 6 females, CASENT 2137987-2137990 (CASC); OSUC 250665 (CNCI). SOUTH AFRICA: 3 females, 13 males, BMNH(E)#790187-790189, 790196-790197, 790199, 848507-848509 (BMNH); OSUC 207935-207937, 207939, 207942, 207947 (CNCI); OSUC 230254 (OSUC). ZIMBABWE: 3 females, 1 male, OSUC 250653-250656 (CNCI).

#### Comments.

Some specimens show variability in the prominence of the sculpture between inner orbit and central keel on the frons.

### Platyscelio pulchricornis

urn:lsid:zoobank.org:act:7290F370-068F-458B-86BB-22CFEB2C2926

urn:lsid:biosci.ohio-state.edu:osuc_concepts:5091

[Fig Figs25-30] [Fig Figs31-36] [Fig Figs37-42] [Fig Figs43-48] [Fig Figs49-54] Figures 25-60 Morphbank^34^

Platyscelio pulchricornis Kieffer 1905: 13 (original description); Kieffer 1926: 553 (description, keyed); Bin 1974: 458 (type information); Kononova and Kozlov 2008: 319 (description).
Platyscelio abnormis Crawford 1910: 126 (original description), syn. n.; Kieffer 1926: 553, 554 (description, keyed); Yasumatsu 1941: 76 (description, synonymy); Masner and Muesebeck 1968: 42 (type information); Lê 2000: 149 (description); Rajmohana K. 2006: 128 (description).
Platyscelio dunensis Mukerjee 1993: 78 (original description), syn. n.
Platyscelio mirabilis Dodd 1913: 132 (original description), syn. n.; Dodd 1915: 444 (description); Kieffer 1926: 553, 554 (description, keyed); Galloway 1976: 101 (type information); Galloway and Austin 1984: 75 (description).
Platyscelio punctatus Kieffer 1913: 321 (original description), syn. n.; Kieffer 1926: 553, 555 (description, keyed); Mani 1936: 337 (variation); Kelner-Pillault 1958: 151 (type information, error).
Platyscelio wilcoxi   Fullaway 1913: 283 (original description); Kieffer 1926: 553, 555 (description, keyed); Yasumatsu 1941: 76 (junior synonym of Platyscelio abnormis Crawford); Masner and Muesebeck 1968: 42 (type information).

#### Description.

##### General:

Body length of male: 3.24–4.71 mm (n=20). Body length of female: 3.31–5.59 mm (n=20).

##### Head:

Length between anterior ocellus and posterior ocellar line in frontal view: less than 0.5 times POL. Striae within ocellar triangle: dense (greater than 20). Vertex sculpture between inner orbit and posterior ocellus: smooth or with few faint striae. Frontal sculpture between inner orbit and central keel: smooth. Submedial ventral area of head anterior to fossa: smooth, finely longitudinally striate posteriorly. Orbital carina: absent. Sculpture of malar region: smooth, longitudinally striate or with few faint striae. Color of female antenna: dark brown to black throughout, A1–A7 yellow to light brown, A8–A12 dark brown to black, or A1–A7 brown, A8–A12 dark brown to black, in some antennae sequentially darker from scape to apex. Female outer lateral apex of scape: sharply pointed. Claval shape: apical margin of A9–A11 concave, closely fitting basal margin of following antennomere. Color of male antenna: brown.

##### Mesosoma:

Sculpture on medial lobe of mesoscutum: longitudinally striate with elongate punctures. Setation of medial lobe of mesoscutum: moderately dense, even. Notaulus: present. Notaulus form: mesal and lateral margin arched. Length of notaulus: percurrent or nearly so, or abbreviated, clearly not reaching anterior margin of mesoscutum. Width of notaulus anteriorly: parallel-sided. Pilosity of notaulus: absent. Number of lateral carinae on mesoscutum: 0. Medial carina of mesoscutum: absent. Parapsidal line: present or absent or faint. Posterior scutellar sulcus: interrupted medially. Setation of posterior half of ventral metapleural area: dense (more than 25 setae). Metascutellum size: wide, metanotum lateral to metascutellum reduced, with 0–3 foveae. Sculpture on ventral metapleural area: smooth to faintly longitudinally striate, or longitudinally striate or with few reticulations. Median propodeal sulcus: narrow throughout length, widened posteriorly. Sculpture of submedian propodeal field: smooth throughout or with few faint striae or longitudinally striate. Posterolateral margin of propodeum: smooth laterally, longitudinally striate to rugulose posteriorly. Color of legs: coxae dark brown to black, otherwise brown, or coxae dark brown to black, otherwise yellow throughout.

##### Wings;

Postmarginal vein: absent. Fore wing: hyaline, infuscate.

##### Metasoma:

Sculpture on T1: longitudinally striate laterally, uniformly setigerous punctate medially. Sublateral carina on T2–T4: present, percurrent. Sculpture on T2–T4: uniformly setigerous punctate.

**Figures 25–30. Figs25-30:**
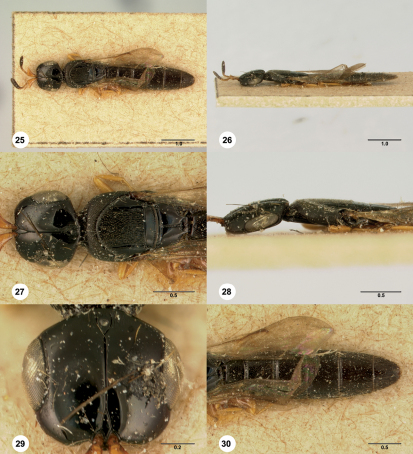
^84^ Platyscelio pulchricornis Kieffer, holotype female (MCSN 0004). 25 Dorsal habitus 26 Lateral habitus 27 Head and mesosoma, dorsal view 28 Head and mesosoma, lateral view 29 Head, dorsal view 30 Metasoma, dorsal view. Scale bars in millimeters.

**Figures 31–36. Figs31-36:**
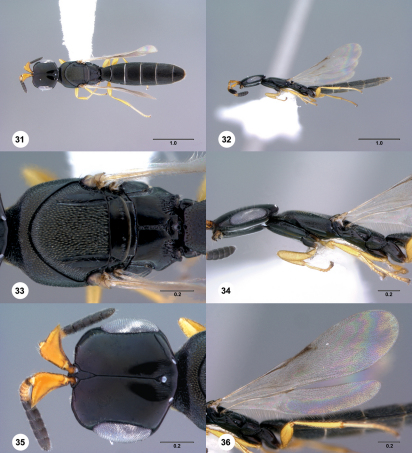
^85^ Platyscelio pulchricornis Kieffer, female (OSUC 207837). 31 Dorsal habitus 32 Lateral habitus 33 Mesosoma, dorsal view 34 Head and mesosoma, lateral view 35 Head, dorsal view 36 Fore and hind wing, lateral view. Scale bars in millimeters.

**Figures 37–42. Figs37-42:**
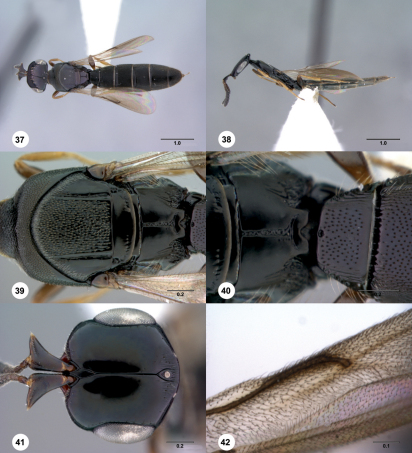
^86^ Platyscelio pulchricornis Kieffer, female (OSUC 250628). 37 Dorsal habitus 38 Lateral habitus 39 Mesosoma, dorsal view 40 Propodeum and T1, dorsal view 41 Head, dorsal view 42 Fore wing, dorsal view. Scale bars in millimeters.

**Figures 43–48. Figs43-48:**
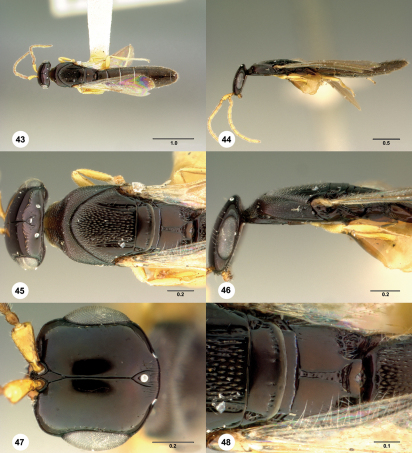
^87^ Platyscelio abnormis Crawford, holotype male (USNM Type No. 12895). 43 Dorsal habitus 44 Lateral habitus 45 Head and mesosoma, dorsal view 46 Head and mesosoma, lateral view 47 Head, dorsal view 48 Mesoscutellum and propodeum, dorsal view. Scale bars in millimeters.

**Figures 49–54. Figs49-54:**
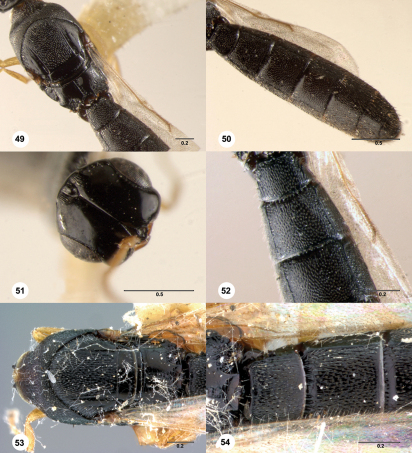
^88^ 49–52 Platyscelio dunensis Mukerjee, holotype male (NZSI 0001). 49 Mesosoma and propodeum, dorsal view 50 Metasoma, dorsal view 51 Head, dorsal view 52 T1 and T2, dorsal view. 53–54 Platyscelio punctatus Kieffer^89^, syntype male (OSUC 207855). 53 Mesosoma, dorsal view 54 T1 and T2, dorsal view. Scale bars in millimeters.

**Figures 55–60.**
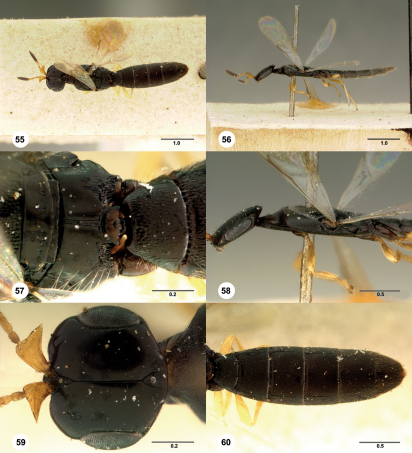
^90^ Platyscelio wilcoxi Fullaway, holotype female (USNM Type No. 26186). 55 Dorsal habitus 56 Lateral habitus 57 Propodeum and T1, dorsal view 58 Head and mesosoma, lateral view 59 Head, dorsal view 60 Metasoma, dorsal view. Scale bars in millimeters.

#### Diagnosis.

Platyscelio pulchricornis can be separated from Platyscelio africanus by the medial absence of the posterior scutellar sulcus and the smoothness of submarginal propodeal field (Figs 33, 39, 45, 48).

#### Link to distribution map. ^35^

#### Material examined.

Holotype female, Platyscelio pulchricornis: PAPUA NEW GUINEA: Madang Prov., Dilo, VI.1890–VII.1890, none specified, Loria, MCSN 0004 (deposited in MCSN). Holotype male, Platyscelio abnormis:PHILIPPINES: R.E. Brown, (deposited in USNM Cat. No. 12895). Holotype male, Platyscelio dunensis: INDIA: Uttarakhand, Rishiskesh, 24.VI.1991, Mukerjee & party (deposited in NZSI). Holotypefemale, Platyscelio mirabilis: AUSTRALIA: Queensland, Nelson, 16.II.1912, sweeping open forest (deposited in SAMA). Syntype male, Platyscelio punctatus: PHILIPPINES: Los Baños (deposited in USNM). Holotype male, Platyscelio wilcoxi: GUAM: USNM No. 26186 (deposited in USNM).

Other material: (121 females, 71 males, 6 unknowns) AUSTRALIA: 1 female, 4 males, BMNH(E)#790202-790203, 790208 (BMNH); OSUC 250637 (CNCI); OSUC 141949 (OSUC). BANGLADESH: 1 female, OSUC 173855 (CNCI). CHINA: 2 females, 4 males, BMNH(E)#848537-848540 (BMNH); OSUC 321841-321842 (CNCI). GUAM: 3 females, 1 male, 3 unknowns, OSUC 250638 (CNCI); OSUC 207850-207852, 207856-207858 (USNM). INDIA: 7 females, 2 males, BMNH(E)#790185, 790206-790207, 848518-848519 (BMNH); OSUC 230648 (OSUC); OSUC 207838-207840 (USNM). INDONESIA: 13 females, 9 males, BMNH(E)#790210 (BMNH); OSUC 207859-207874, 207876, 207893-207894, 207896 (CNCI); OSUC 204851 (UCDC). JAPAN: 1 female, OSUC 173082 (UASK). MALAYSIA: 9 females, 5 males, BMNH(E)#790193, 790204 (BMNH); OSUC 207877-207883, 207885-207887, 207892 (CNCI); OSUC 207884 (SCAU). PAPUA NEW GUINEA: 24 females, 21 males, BMNH(E)#790205, 848511-848517 (BMNH); CASENT 2137992-2138022 (CASC); OSUC 207897, 250626-250628 (CNCI); OSUC 160037-160038 (EMEC). PHILIPPINES: 3 females, 7 males, BMNH(E)#790184 (BMNH); OSUC 207875 (CNCI); OSUC 207842-207849 (USNM). SOLOMON ISLANDS: 1 female, BMNH(E)#848543 (BMNH). TAIWAN: 18 females, 11 males, OSUC 173803-173831 (TARI). THAILAND: 36 females, 2 males, 2 unknowns, BMNH(E)#790190, 790192, 790194, 848502-848505, 848541-848542 (BMNH); OSUC 207888-207890 (CNCI); OSUC 207837, 215797, 253701-253721, 253760-253762, 253764 (OSUC); OSUC 207891 (SCAU). VANUATU: 2 males, 1 unknown, BMNH(E)#790183, 790186, 790198 (BMNH). VIETNAM: 2 females, 3 males, OSUC 277708-277709 (RMNH); OSUC 184378-184380 (ZMAS).

#### Comments.

Some specimens show variability in antenna and leg color. Because of the uniformity of the principal characters – notaulus form and length, interrupted posterior scutellar sulcus, and the propodeal sculpture – we consider these specimens to be conspecific with Platyscelio pulchricornis.

### Platyscelio striga

Taekul & Johnson sp. n.

urn:lsid:zoobank.org:act:A1051E41-5E69-4792-8C30-7B2F928E56A8

urn:lsid:biosci.ohio-state.edu:osuc_concepts:242614

**Figures 61–66.**
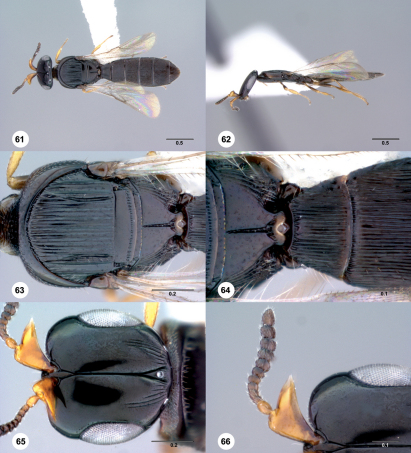
^91^ Platyscelio striga, sp. n., holotype female (OSUC 250630). 61 Dorsal habitus 62 Lateral habitus 63 Mesosoma, dorsal view 64 Propodeum and T1, dorsal view 65 Head, dorsal view 66 Antennae, dorsal view. Scale bars in millimeters.

Figures 61–66 Morphbank^36^ 

#### Description

##### General:

Body length of male: 3.05–3.22 mm (n=2). Body length of female: 2.89–3.40 mm (n=2).

##### Head:

Length between anterior ocellus and posterior ocellar line in frontal view: less than 0.5 times POL. Striae within ocellar triangle: dense (greater than 20). Vertex sculpture between inner orbit and posterior ocellus: smooth or with few faint striae. Frontal sculpture between inner orbit and central keel: with few (4–5) striae, striae limited to upper half of frons. Submedial ventral area of head anterior to fossa: longitudinally striate throughout. Orbital carina: absent. Sculpture of malar region: longitudinally striate or with few faint striae.

##### Antenna:

Color of female antenna: A1–A7 brown, A8–A12 dark brown to black, in some antennae sequentially darker from scape to apex. Female outer lateral apex of scape: sharply pointed. Claval shape: apical margin of A9–A11 straight, antennomeres distinctly separated. Color of male antenna: dark brown to black throughout.

##### Mesosoma:

Sculpture on medial lobe of mesoscutum: longitudinally striate. Setation of medial lobe of mesoscutum: sparse to glabrous. Notaulus: present. Notaulus form: mesal margin arched, lateral margin straight. Length of notaulus: abbreviated, clearly not reaching anterior margin of mesoscutum. Width of notaulus anteriorly: narrowed anteriorly. Pilosity of notaulus: absent. Number of lateral carinae on mesoscutum: 2. Medial carina of mesoscutum: absent. Parapsidal line: absent or faint. Posterior scutellar sulcus: complete. Setation of posterior half of ventral metapleural area: sparse (less than 25 setae). Metascutellum size: wide, metanotum lateral to metascutellum reduced, with 0–3 foveae. Sculpture on ventral metapleural area: strongly reticulate rugose with foveolae. Median propodeal sulcus: narrow throughout length. Sculpture of submedian propodeal field: smooth throughout or with few faint striae. Posterolateral margin of propodeum: longitudinally striate laterally, rugulose posteriorly. Color of legs: coxae dark brown to black, otherwise yellow in female and brown in male.

##### Wings:

Postmarginal vein: absent. Fore wing: hyaline.

##### Metasoma:

Sculpture on T1: longitudinally striate throughout. Sublateral carina on T2–T4: absent. Sculpture on T2–T4: longitudinally striate throughout, uniformly setigerous punctate within interstices on T3–T4.

#### Diagnosis.

Platyscelio striga is unique in the genus in having two lateral carinae on the mesoscutum (Fig. 63), and no sublateral carinae on T2–T4 (Figs 61, 64).

#### Etymology.

The epithet striga, Latin for swath, refers to the distinct striae of the frons.

#### Link to distribution map. ^37^

#### Material examined.

Holotype female: AUSTRALIA: Western Australia, Cape Arid N.P., 33°31.134'S, 123°26.052'E (WDPA-UN), 30.XII–3.I.1987, Malaise trap, J.S. Noyes, OSUC 250630 (deposited in ANIC). Paratypes: AUSTRALIA: 1 female, 2 males, OSUC 250629, 250631, 250632 (CNCI).

### Platyscelio mzantsi

Taekul & Johnson sp. n.

urn:lsid:zoobank.org:act:D084EF48-4736-444F-916F-2C8CDE23E29B

urn:lsid:biosci.ohio-state.edu:osuc_concepts:242617

**Figures 67–72.**
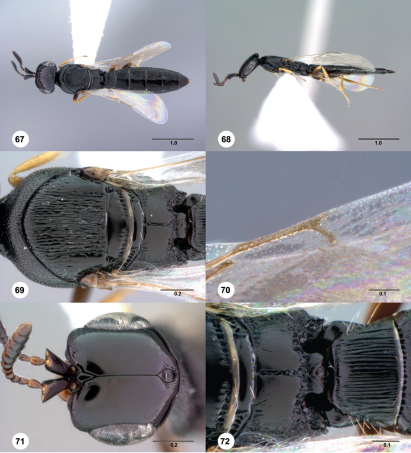
^92^ Platyscelio mzantsi, sp. n., holotype female (OSUC 243790). 67 Dorsal habitus 68 Lateral habitus 69 Mesosoma, dorsal view 70 Fore wing marginal vein, dorsal view 71 Head, dorsal view 72 Propodeum and T1, dorsal view. Scale bars in millimeters.

Figures 67–72 Morphbank^38^ 

#### Description.

##### General:

Body length of male: 3.21–4.53 mm (n=20). Body length of female: 3.14–4.23 mm (n=20).

##### Head:

Length between anterior ocellus and posterior ocellar line in frontal view: greater than or equal to 0.5 times POL. Striae within ocellar triangle: sparse (equal to or less than 20). Vertex sculpture between inner orbit and posterior ocellus: smooth or with few faint striae. Frontal sculpture between inner orbit and central keel: smooth. Submedial ventral area of head anterior to fossa: longitudinally striate throughout. Orbital carina: absent. Sculpture of malar region: longitudinally striate or with few faint striae.

##### Antenna:

Color of female antenna: dark brown to black throughout. Female outer lateral apex of scape: bluntly rounded. Claval shape: apical margin of A9–A11 concave, closely fitting basal margin of following antennomere. Color of male antenna: dark brown to black throughout.

##### Mesosoma:

Sculpture on medial lobe of mesoscutum: longitudinally striate. Setation of medial lobe of mesoscutum: moderately dense, even. Notaulus: present. Notaulus form: mesal and lateral margin arched. Length of notaulus: percurrent or nearly so. Width of notaulus anteriorly: parallel-sided. Pilosity of notaulus: absent. Number of lateral carinae on mesoscutum: 0. Medial carina of mesoscutum: absent. Parapsidal line: present or absent or faint. Posterior scutellar sulcus: interrupted medially. Setation of posterior half of ventral metapleural area: sparse (less than 25 setae). Metascutellum size: narrow, metanotum lateral to metascutellum with 4–6 foveae. Sculpture on ventral metapleural area: longitudinally striate or with few reticulations. Median propodeal sulcus: narrow throughout length. Sculpture of submedian propodeal field: longitudinally striate. Posterolateral margin of propodeum: margined by coarsely punctate furrow. Color of legs: coxae dark brown to black, otherwise brown.

**Figures 73–76.**
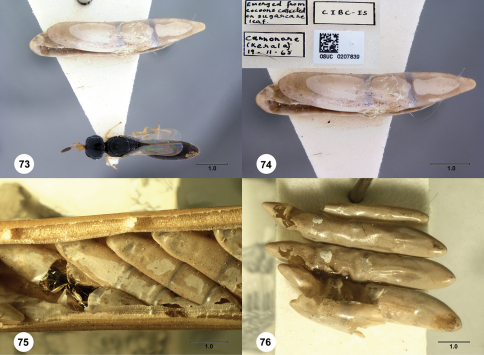
^93^ Platyscelio host eggs. 73 Platyscelio pulchricornis and host egg collected from sugarcane, dorsal view (OSUC 207839) 74 Host egg and specimen label (OSUC 207839) 75 Platyscelio pulchricornis host eggs collected from rice (BMNH(E)#790194) 76 Host eggs collected from sugarcane (BMNH(E)#790205). Scale bars in millimeters

##### Wings:

Postmarginal vein: reduced, stumplike. Fore wing: hyaline or infuscate.

##### Metasoma:

Sculpture on T1: longitudinally striate throughout. Sublateral carina on T2–T4: present, percurrent. Sculpture on T2–T4: T2 longitudinally striate throughout, T3–T4 longitudinally reticulate laterally, smooth medially.

#### Diagnosis.

Platyscelio mzantsi may be separated from other African species by the sparse striae within ocellar triangle (20 or fewer), the narrow metascutellum, and the presence of 4–6 foveae on the metanotum laterad of the metascutellum (Figs 71, 72). Some specimens have the sculpture on the submedian propodeal field strongly effaced.

#### Etymology.

The epithet mzantsi, Xhosa for south, is a reference to the the collecting locality.

#### Link to distribution map. ^39^

#### Material examined.

Holotype female: SOUTH AFRICA: 34°27.414'S 19°21.393'E Western Cape, Walker Bay Nat. Res., 57 m, Site1, Malaise trap, S. Coast Strandveld, 4.X–1.XI.1997, S.van Noort, WB97–M11, OSUC 243790 (deposited in SAMC). Paratypes: SOUTH AFRICA: 63 females, 31 males, OSUC 202440–202441 (AEIC); OSUC 207908–207934, 207941, 250662, 250664 (CNCI); OSUC 266101–266102 (MZLU); OSUC 188488–188489, 207830–207836, 207898–207907, 207940, 213995, 226020–226023, 237213–237217, 243454–243458, 243506–243507, 243790–243791, 253729–253740, 253745–253753 (SAMC).

## Supplementary Material

XML Treatment for Platyscelio

## Endnotes

1 http://biocol.org/urn:lsid:biocol.org:col:1008

2 http://biocol.org/urn:lsid:biocol.org:col:32981

3 http://biocol.org/urn:lsid:biocol.org:col:1009

4 http://biocol.org/urn:lsid:biocol.org:col:33071

5 http://biocol.org/urn:lsid:biocol.org:col:1012

6 http://biocol.org/urn:lsid:biocol.org:col:1013

7 http://biocol.org/urn:lsid:biocol.org:col:33557

8 http://biocol.org/urn:lsid:biocol.org:col:33785

9 http://biocol.org/urn:lsid:biocol.org:col:33943

10 http://biocol.org/urn:lsid:biocol.org:col:34072

11 http://biocol.org/urn:lsid:biocol.org:col:1014

12 http://biocol.org/urn:lsid:biocol.org:col:34212

13 http://biocol.org/urn:lsid:biocol.org:col:1017

14 http://biocol.org/urn:lsid:biocol.org:col:34982

15 http://biocol.org/urn:lsid:biocol.org:col:1018

16 http://biocol.org/urn:lsid:biocol.org:col:34248

17 http://biocol.org/urn:lsid:biocol.org:col:34252

18 http://biocol.org/urn:lsid:biocol.org:col:34337

19 http://biocol.org/urn:lsid:biocol.org:col:34403

20 http://biocol.org/urn:lsid:biocol.org:col:34411

21 http://biocol.org/urn:lsid:biocol.org:col:1019

22 http://biocol.org/urn:lsid:biocol.org:col:34593

23 http://biocol.org/urn:lsid:biocol.org:col:34624

24 http://vsyslab.osu.edu

25 http://specimage.osu.edu

26 http://www.tdwg.org

27 http://hol.osu.edu/map-large.html?id=543&view=all

28 http://www.morphbank.net/?id=515106

29 http://hol.osu.edu/map-large.html?id=5089

30 http://www.morphbank.net/?id=514554

31 http://hol.osu.edu/map-large.html?id=242615

32 http://www.morphbank.net/?id=514555

33 http://osuc.biosci.ohio-state.edu/HymOnline/map-large.html?id=242616

34 http://www.morphbank.net/?id=515112

35 http://hol.osu.edu/map-large.html?id=5091

36 http://www.morphbank.net/?id=514563

37 http://hol.osu.edu/map-large.html?id=242614

38 http://www.morphbank.net/?id=514564

39 http://hol.osu.edu/map-large.html?id=242617

40 http://lsid.tdwg.org/urn:lsid:biosci.ohio-state.edu:osuc_pubs:20940

41 http://lsid.tdwg.org/urn:lsid:biosci.ohio-state.edu:osuc_pubs:21201

42 http://lsid.tdwg.org/urn:lsid:biosci.ohio-state.edu:osuc_pubs:119

43 http://lsid.tdwg.org/urn:lsid:biosci.ohio-state.edu:osuc_pubs:127

44 http://lsid.tdwg.org/urn:lsid:biosci.ohio-state.edu:osuc_pubs:131

45 http://lsid.tdwg.org/urn:lsid:biosci.ohio-state.edu:osuc_pubs:175

46 http://lsid.tdwg.org/urn:lsid:biosci.ohio-state.edu:osuc_pubs:197

47 http://lsid.tdwg.org/urn:lsid:biosci.ohio-state.edu:osuc_pubs:623

48 http://lsid.tdwg.org/urn:lsid:biosci.ohio-state.edu:osuc_pubs:343

49 http://lsid.tdwg.org/urn:lsid:biosci.ohio-state.edu:osuc_pubs:436

50 http://lsid.tdwg.org/urn:lsid:biosci.ohio-state.edu:osuc_pubs:353

51 http://lsid.tdwg.org/urn:lsid:biosci.ohio-state.edu:osuc_pubs:339

52 http://lsid.tdwg.org/urn:lsid:biosci.ohio-state.edu:osuc_pubs:10850

53 http://lsid.tdwg.org/urn:lsid:biosci.ohio-state.edu:osuc_pubs:229

54 http://lsid.tdwg.org/urn:lsid:biosci.ohio-state.edu:osuc_pubs:21725

55 http://lsid.tdwg.org/urn:lsid:biosci.ohio-state.edu:osuc_pubs:327

56 http://lsid.tdwg.org/urn:lsid:biosci.ohio-state.edu:osuc_pubs:418

57 http://lsid.tdwg.org/urn:lsid:biosci.ohio-state.edu:osuc_pubs:308

58 http://lsid.tdwg.org/urn:lsid:biosci.ohio-state.edu:osuc_pubs:309

59 http://lsid.tdwg.org/urn:lsid:biosci.ohio-state.edu:osuc_pubs:323

60 http://lsid.tdwg.org/urn:lsid:biosci.ohio-state.edu:osuc_pubs:402

61 http://lsid.tdwg.org/urn:lsid:biosci.ohio-state.edu:osuc_pubs:310

62 http://lsid.tdwg.org/urn:lsid:biosci.ohio-state.edu:osuc_pubs:22163

63 http://lsid.tdwg.org/urn:lsid:biosci.ohio-state.edu:osuc_pubs:321

64 http://lsid.tdwg.org/urn:lsid:biosci.ohio-state.edu:osuc_pubs:9718

65 http://lsid.tdwg.org/urn:lsid:biosci.ohio-state.edu:osuc_pubs:794

66 http://lsid.tdwg.org/urn:lsid:biosci.ohio-state.edu:osuc_pubs:219

67 http://lsid.tdwg.org/urn:lsid:biosci.ohio-state.edu:osuc_pubs:236

68 http://lsid.tdwg.org/urn:lsid:biosci.ohio-state.edu:osuc_pubs:311

69 http://lsid.tdwg.org/urn:lsid:biosci.ohio-state.edu:osuc_pubs:474

70 http://lsid.tdwg.org/urn:lsid:biosci.ohio-state.edu:osuc_pubs:312

71 http://lsid.tdwg.org/urn:lsid:biosci.ohio-state.edu:osuc_pubs:21300

72 http://lsid.tdwg.org/urn:lsid:biosci.ohio-state.edu:osuc_pubs:240

73 http://lsid.tdwg.org/urn:lsid:biosci.ohio-state.edu:osuc_pubs:21324

74 http://lsid.tdwg.org/urn:lsid:biosci.ohio-state.edu:osuc_pubs:21274

75 http://www.mapress.com/zootaxa/2008/f/zt01671p031.pdf

76 http://lsid.tdwg.org/urn:lsid:biosci.ohio-state.edu:osuc_pubs:21131

77 http://lsid.tdwg.org/urn:lsid:biosci.ohio-state.edu:osuc_pubs:1000

78 http://mapress.com/zootaxa/2007f/zt01668p548.pdf

79 http://lsid.tdwg.org/urn:lsid:biosci.ohio-state.edu:osuc_pubs:829

80 http://www.morphbank.net/?id=514542

81 http://www.morphbank.net/?id=514543

82 http://www.morphbank.net/?id=514554

83 http://www.morphbank.net/?id=514555

84 http://www.morphbank.net/?id=514556

85 http://www.morphbank.net/?id=514557

86 http://www.morphbank.net/?id=514558

87 http://www.morphbank.net/?id=514559

88 http://www.morphbank.net/?id=514560

89 http://www.morphbank.net/?id=514561

90 http://www.morphbank.net/?id=514562

91 http://www.morphbank.net/?id=514563

92 http://www.morphbank.net/?id=514564

93 http://www.morphbank.net/?id=515025
